# High-resolution analog-to-digital converter-based data acquisition system for geoelectrical prospecting

**DOI:** 10.1016/j.ohx.2026.e00837

**Published:** 2026-09-09

**Authors:** Miguel López Jiménez, Alfredo Ghisays Ruiz, Paola Pacheco Martínez, Juan Álvarez Navarro

**Affiliations:** Physics Program, Universidad del Atlántico, Barranquilla, Colombia

**Keywords:** Electrical resistivity, ADC, Geophysical instrumentation, Data acquisition

## Abstract

Electrical resistivity surveying is a fundamental geophysical technique for subsurface characterization, based on the measurement of apparent resistivity using the four-point method. The reliable acquisition of these data requires high-resolution and stable instrumentation, capable of detecting small variations in electrical signals.

This article presents the development of a low-cost, high-resolution data acquisition system based on a 24-bit analog-to-digital converter (ADC), which enables the recording of injected currents and resulting voltages in the subsurface. The system incorporates automatic polarity reversal, allowing the discrimination of spontaneous potential and improving the quality of the measurements.

The system was validated through laboratory tests of linearity, repeatability, temporal stability, and dynamic range, using precision resistors and comparing the results with a commercial resistivity meter. The results demonstrate stable and reproducible performance, with the capability to measure low-magnitude signals, making the system suitable for electrical resistivity surveying applications.

## Graphical abstract

**Figure:**
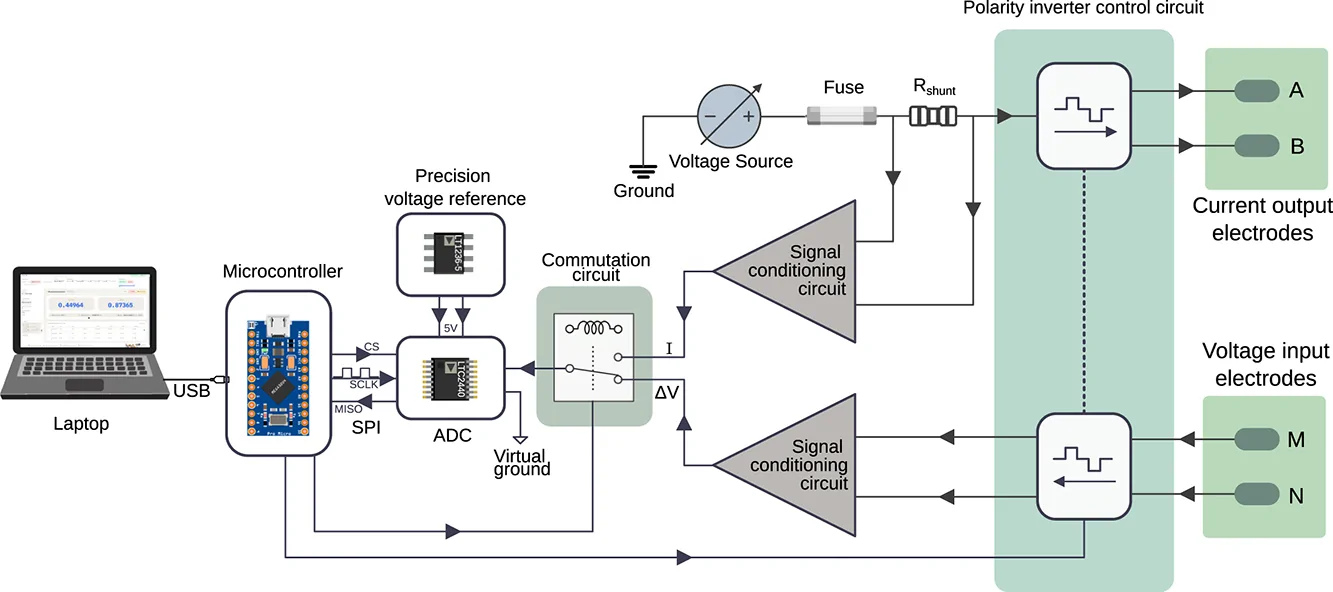

## Specifications table

**Table:** 

Hardware name	*PRISM (Precision Resistivity Instrument for Subsurface Measurements)*
Subject area	*Environmental, planetary and agricultural sciences.*
Hardware type	*Measuring physical properties and in-lab sensors*

Closest commercial analog	***PASI Earth Resistivity Meter MOD. 16GL-N**. PRISM is intended as an alternative to this instrument for four-electrode electrical resistivity surveys. Compared with the PASI system, SERM employs a 24-bit ADC instead of a 16-bit ADC, providing higher acquisition resolution. In addition, while the PASI instrument is operated through an onboard console and physical buttons, PRISM is controlled through a graphical user interface, allowing measurement control, data storage, and data export directly from the software.*

Open source license	*Software license: GPL-3.0, Hardware license: CERN-OHL-S-2.0*
Cost of hardware	*$360.22 USD for all electronic components and $204.95 USD for the enclosure.*
Source file repository	*https://doi.org/10.17605/OSF.IO/AUPMJ*

## 1 Hardware in context

Geoelectrical surveying is one of the most widely used geophysical techniques for subsurface characterization due to its ability to provide non-invasive information on the spatial distribution of electrical resistivity. These systems have applications in a wide range of fields, including groundwater exploration and management, environmental investigations for the detection of contamination and leachates, civil engineering for the geotechnical characterization of foundations and infrastructure works, mineral exploration, dam and levee monitoring, the study of geological and archaeological processes, as well as the temporal monitoring of dynamic processes through electrical resistivity tomography (ERT) and time-lapse measurements. In academia, they also constitute a fundamental tool for research in applied geophysics, the development of new acquisition and inversion methodologies, and the practical training of students and researchers.

The development of these applications has been closely linked to the evolution of geoelectrical instrumentation, from traditional single-channel configurations to multi-electrode and multichannel systems capable of performing 2D, 3D, and time-lapse acquisitions with greater efficiency. In this context, commercial instruments have prioritized robustness, automation, and field acquisition capabilities; however, their high cost, the limited possibility of modifying their architecture, and the difficulty of adapting them to specific experimental requirements continue to represent significant constraints for numerous research groups. In parallel, open-hardware and low-cost initiatives such as OhmPi [1], together with recent developments of autonomous electrical resistivity monitoring systems based on low-cost instrumentation and open-source processing tools, demonstrate a growing trend toward more accessible, reproducible, and customizable solutions.

Within this context, the hardware presented in this work is designed for the acquisition of injected currents and potential differences in four-electrode geoelectrical configurations. The system incorporates an automatic polarity reversal mechanism to reduce the influence of spontaneous potentials and other unwanted contributions to the measurements. This approach is consistent with direct-current measurement protocols that compare the responses obtained under forward and reverse current injection to discriminate spurious signal components. Furthermore, the integration of automatic switching with high-resolution analog-to-digital conversion aims to improve repeatability, stability, and data quality in scenarios where the signals of interest are of very low amplitude and are affected by electrical noise, contact imbalance, and environmental variations.

## 2 Hardware description

PRISM is an open data acquisition system for geoelectrical surveying based on the four-electrode method, specifically designed for the measurement of injected currents and resulting voltages during subsurface electrical resistivity surveys. The system integrates a high-resolution data acquisition stage, analog signal conditioning circuits, automatic polarity reversal, and a graphical user interface (GUI), implemented using web technologies (HTML, CSS, and JavaScript), for controlling the acquisition process.

The acquisition hardware is controlled by an ATmega32U4 microcontroller, which coordinates the operation of the different electronic subsystems. This device manages relay switching, the reversal of the injected current polarity (to discriminate the spontaneous potential of the ground), and the acquisition of signals from the measurement circuits.

**Fig. 1:**
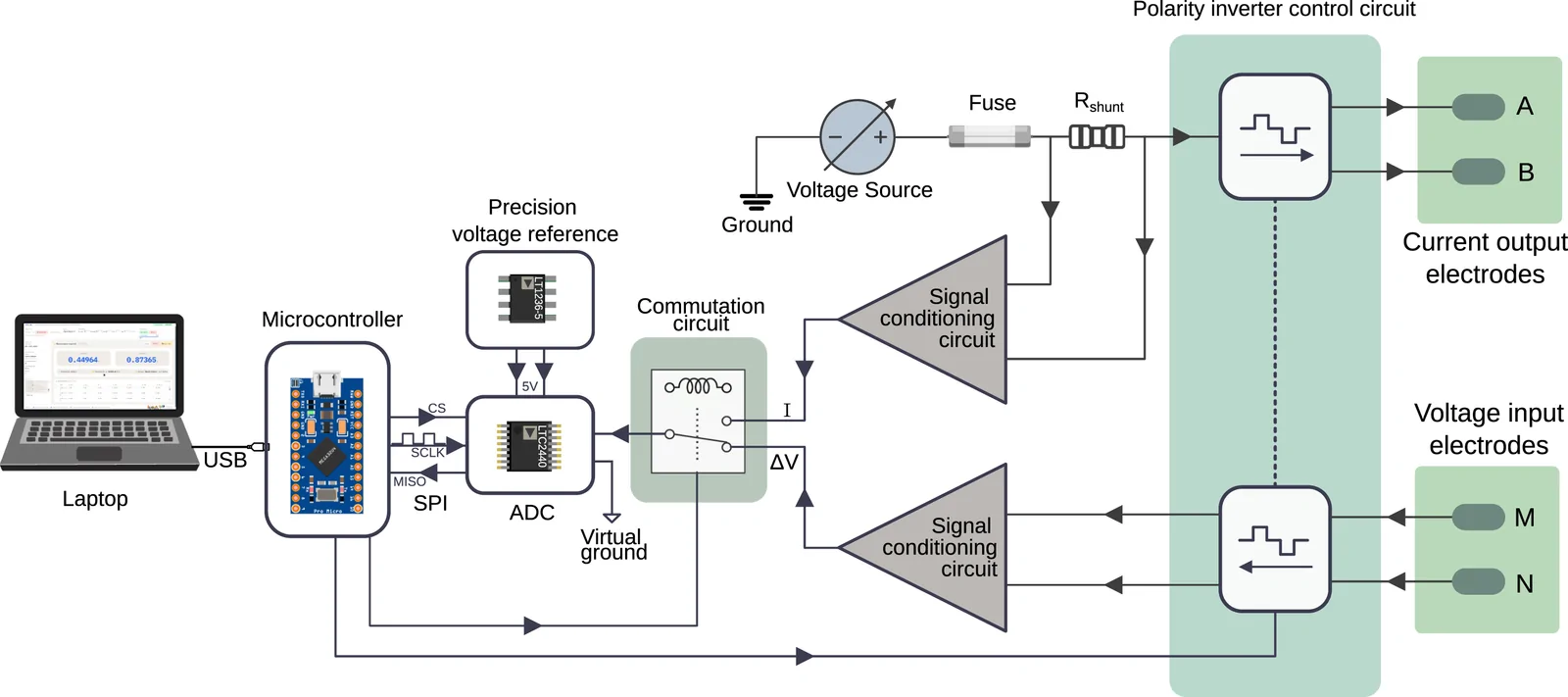
Block diagram of the implemented control and data acquisition architecture, showing the interaction between the user, the software, and the hardware.

Fig. 1 presents the functional block diagram of the developed system. The instrument consists of a adjustable voltage source and a switching circuit responsible for reversing the polarity of both the current injection electrodes (A and B) and the potential measurement electrodes (M and N). The injected current and the measured potential difference are conditioned by dedicated analog circuits before being digitized by a 24-bit analog-to-digital converter (LTC2440), using a high-stability voltage reference (LT1236-5) and a virtual ground to maximize measurement accuracy. Finally, a microcontroller coordinates the acquisition sequence, processes the data, and transmits them to a computer for visualization and storage.

The use of a 24-bit ADC provides high measurement resolution, which is particularly relevant in geoelectrical studies, where both the injected currents and the measured potential differences may exhibit small variations. This resolution enables more accurate recording of changes in current and electrical potential, reducing quantization effects and improving the system is ability to resolve fine variations in the measured signals. This contributes to a more reliable estimation of apparent resistivity and higher quality of the acquired data.

The developed system is capable of measuring currents in the range from 100μA to 100mA. Likewise, it can measure potential differences in the ranges from −2V to −100μV and from 100μV to 10V, allowing it to adapt to both very low-amplitude signals and operating conditions that require higher excitation levels.

### 2.1 Electrical resistivity surveying

Electrical resistivity surveying using direct current is a geophysical exploration method based on an artificially generated steady field, in which an electrical current is injected into the subsurface through a pair of electrodes, referred to as A-B, placed on the ground surface, while the electrical response (voltage) of the subsurface is measured using another pair of electrodes, M-N. This electrical response provides information about the electrical resistivity of the subsurface at different depths [2].

**Fig. 2:**
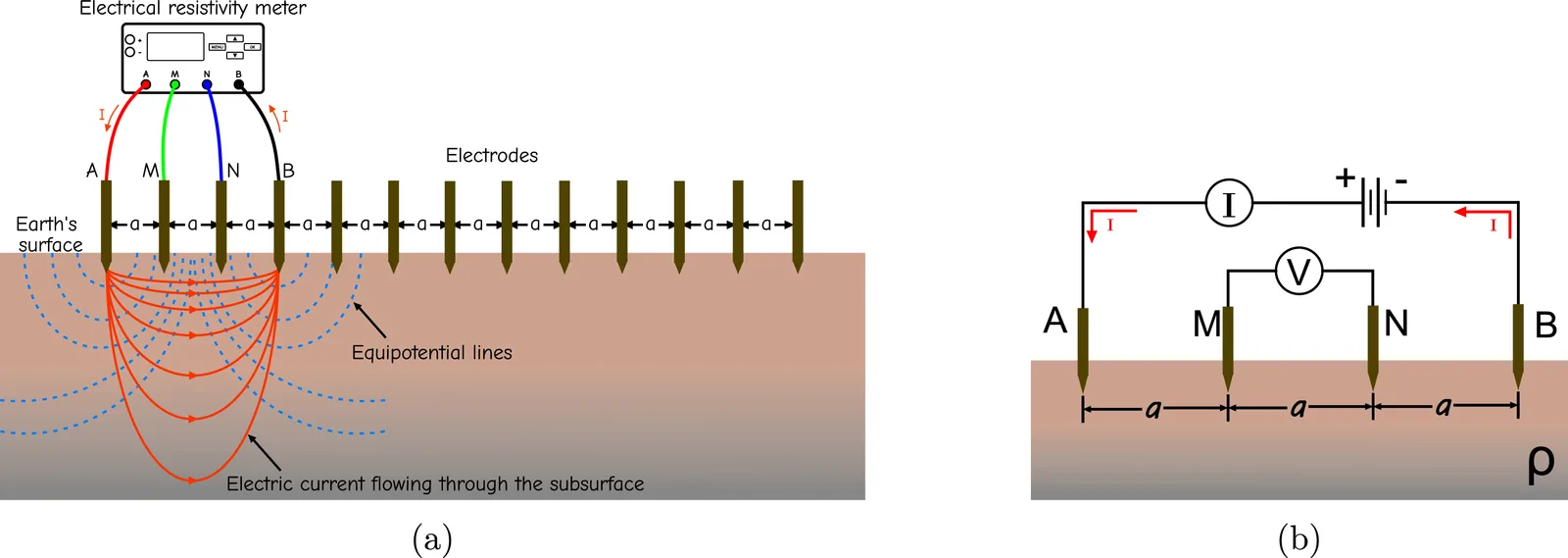
Direct-current electrical resistivity surveying. (a) Basic schematic illustrating the application of the electrical resistivity surveying method. (b)Arrangement of current and potential electrodes in the Wenner configuration.

Fig. 2(a) illustrates an array of electrodes deployed over a homogeneous medium, the propagation of the electric field generated by the injected current, and the equipotential lines resulting from the presence of the field. The apparent resistivity obtained from each measurement corresponds to the midpoint of the four-electrode array AMNB, and the investigation depth depends on the electrode spacing. In other words, as the interelectrode spacing increases, apparent resistivity measurements are obtained from greater depths.

The different ways in which resistivity measurements can be acquired in the field using this method are known as electrode arrays or electrode configurations. These configurations allow the apparent resistivity $\rho_{a}$ (Ωm) of the subsurface to be calculated at a given depth from the geometric factor K (m), the current I (A) injected through electrodes A and B, and the potential difference ΔV (V) measured between electrodes M and N. Each configuration is defined by the spatial arrangement of electrodes A, B, M, and N on the ground surface. The most commonly used electrode configurations are Wenner, Schlumberger, and Dipole-Dipole [3].

The Wenner configuration is characterized by four electrodes aligned along the ground surface with equal spacing a (m) between adjacent electrodes. As shown in Fig. 2(b), the array geometry requires that

(1) $$\bar{AM}=\bar{MN}=\bar{BN}=a,$$

Therefore, the apparent resistivity associated with this array is given by

(2) $$\rho_{a}=2\pi a\frac{\Delta V}{I}.$$

Unlike conventional commercial resistivity meters, whose electronic design and firmware are typically proprietary, the proposed system was conceived under an open-hardware philosophy, allowing the complete modification, adaptation, and reproduction of both its electronic and software components. This feature facilitates its use in both geophysical research and educational environments, where access to commercial instrumentation is often limited by its high cost. Table 1 presents a comparison between the developed system (PRISM), the open-source resistivity meter OhmPi [1], and a commercial PASI resistivity meter [4].

**Fig. 3:**
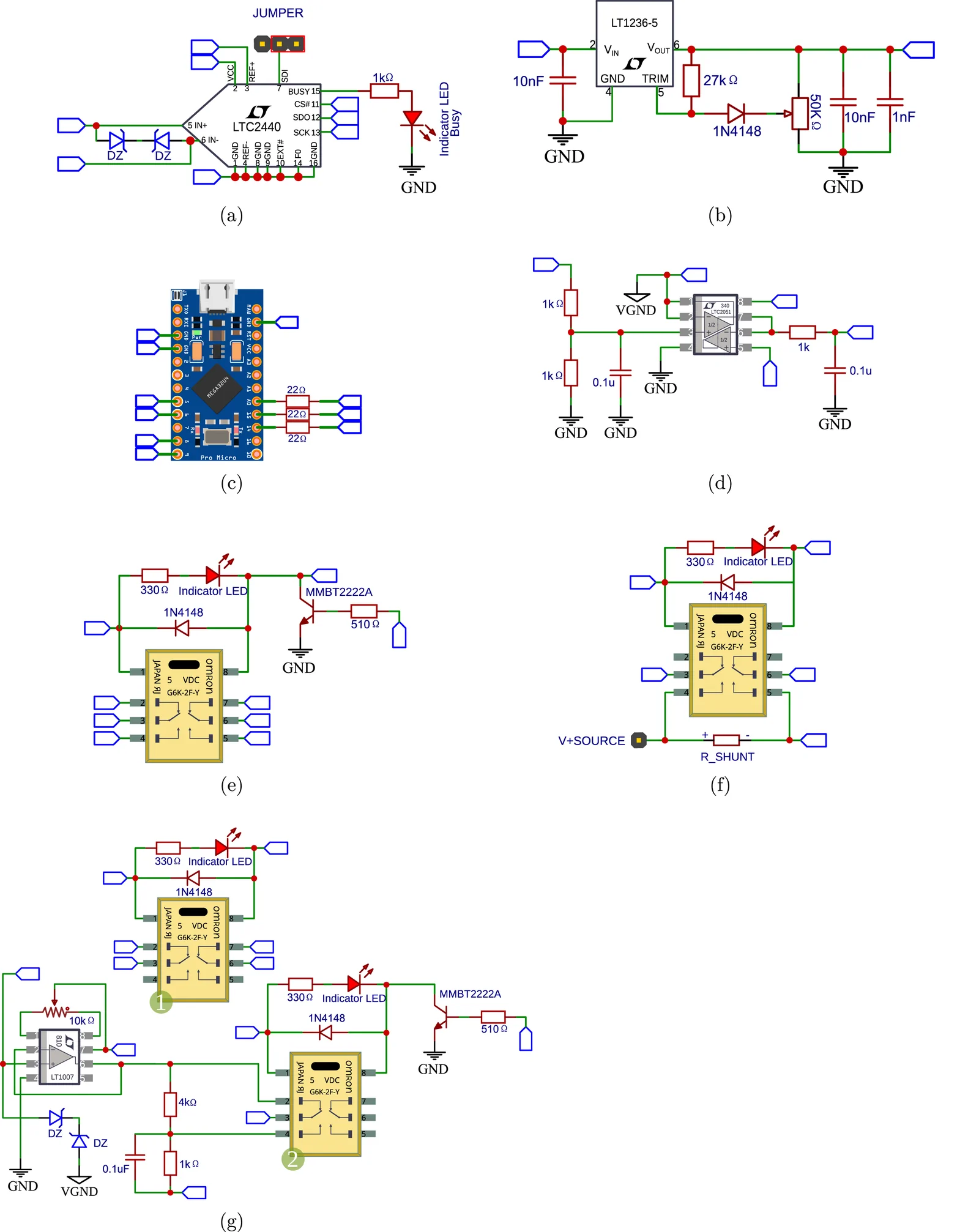
Conditioning and measurement circuit for injected currents and resulting voltages. (a) LTC2440 24-bit analog-to-digital converter. (b) LT1236-5 reference voltage. (c) Arduino Pro Micro board, based on the ATmega32U4 microcontroller. (d) Virtual ground and buffer circuit based on the LTC2051. (e) Selector relay for voltage or current measurement. (f) Shunt resistor enable relay for AB current measurement. (g) MN resulting voltage signal input and conditioning.

The acquisition core is based on the 24-bit LTC2440 analog-to-digital converter (ADC) (Fig. 3(a)), selected for its high resolution and low noise performance [5]. Pins 1 and 2 of the ADC are used for the power supply, while pins 3 and 4 correspond to the reference voltage inputs, which the ADC uses as the reference for comparing and converting the analog input voltages. In this design, the LT1236-5 integrated circuit (Fig. 3(b)) is employed to provide a stable reference voltage of $V_{ref}=5.000\;V$ with low thermal drift [6].

**Table 1:** Comparison between PRISM, OhmPi, and a commercial resistivity meter.

Feature	PRISM	OhmPi	PASI MOD. 16GL-N
ADC	LTC2440 (24-bit)	ADS1115 (16-bit)	16-bit ADC
Nominal ADC resolution	24 bits	16 bits	16 bits
Effective resolution (ENOB)	19-bits	Not reported	Not reported
Voltage range for ${\text{V}}_{\text{MN}}$	from −0.1mV to −2V and from 0.1mV to 10V	from 1mV to 12V	from ±20mV to ±1280V
Current range for ${\text{I}}_{\text{AB}}$	from 0.1mA to 100mA	from 0.1mA to 80mA	Not reported
Maximum measurement error	0.95%	2.07%	Not reported
Automatic polarity reversal	Yes	Yes	Yes
GUI	HTML/CSS/JavaScript	Python-based	No
Embedded display/keypad	No	No	Yes
Open-source hardware	Yes	Yes	No
Data export	Yes	Yes	Instrument software
Approximate hardware cost (USD)	$360.22	<$500	Commercial product

Pins 5 and 6 receive the differential voltage signal from the subsurface, which has been previously conditioned by the analog front-end of the system. The ADC sampling rate is configured through pin 7 (SDI). When this pin is connected to *V_CC_*, a conversion rate of 6.9Hz is established, whereas connecting it to ground (GND) increases the conversion rate to 880Hz. Pins 8, 9, 10, 14, and 16 are connected to 0V, providing the internal reference and stability functions required by the device. Pins 11, 12, and 13 correspond to the SPI digital communication interface, through which the digitized data are transmitted to the microcontroller for subsequent processing and storage. Finally, pin 15 is connected to a status LED that is activated during the conversion process, providing a visual indication of the ADC conversion status.

### 2.2 Control and data acquisition circuit

The system is controlled by an Arduino Pro Micro board based on the ATmega32U4 microcontroller (Fig. 3(c)), which executes the acquisition routines, synchronizes the operation of the different functional blocks, performs ADC readout, basic data processing, and communication with the user interface.

The control of the relays responsible for signal conditioning is distributed among several digital pins of the microcontroller. In particular, the controlled selection of the electrical quantity to be sent to the subsequent digitization stage is performed through the VI_SEL (Voltage/Current Select) pin, which actuates the relay shown in Fig. 3(e). This relay switches between the measurement of the *MN* voltage from the subsurface and the voltage developed across the *shunt* resistor as a result of the injected *AB* current.

### 2.3 Signal conditioning circuits

A virtual ground and buffer circuit (Fig. 3(d)) was implemented, whose primary function is to generate an intermediate reference voltage of 2.5 V, with respect to which the ADC input voltages are measured, thereby enabling the proper conditioning of bipolar signals in a single-supply system.

According to the manufacturer’s datasheet, the ADC is capable of measuring potential differences within the range of $-0.5V_{\text{ref}}$ to $0.5V_{\text{ref}}$, which in this design corresponds to a measurement range from −2.5V to 2.5 V. However, the allowable voltage at the ADC input pins is limited to the interval between GND−0.3V and Vcc+0.3V. Therefore, to enable the measurement of negative potential differences without violating the operating limits of the device, it is necessary to generate a *virtual ground* reference at 2.5 V. This shifts the DC level of the input signal, providing a measurement range that is symmetrical with respect to this reference.

This circuit employs the LTC2051 integrated circuit, which incorporates two operational amplifiers configured as voltage followers, providing low output impedance and adequate signal stability. The first operational amplifier is used as a buffer for the virtual ground, ensuring its stability under varying load conditions, while the second is used as a buffer for the ADC input signal.

### 2.4 Measurement circuit

To condition the signals acquired from the subsurface prior to digitization, while minimizing noise and ensuring compatibility with the ADC, the system incorporates dedicated circuits for voltage measurement (Fig. 3(g)) and current measurement (Fig. 3(f)).

Fig. 3(g) corresponds to the input stage dedicated to measuring the resulting voltage *MN* in the subsurface. The primary function of this stage is to isolate the signal from the ground through relay (1) and to provide a high input impedance by means of an LT1007 operational amplifier configured as a voltage follower. The resulting voltage is applied directly to the non-inverting input (+IN) of the operational amplifier, using the system virtual ground as the reference.

To minimize systematic errors associated with DC offset, a 10kΩ multiturn potentiometer is connected between pins 1 and 8 of the LT1007, allowing the offset voltage to be adjusted and compensated according to the manufacturer’s recommendations [7]. The output of the operational amplifier is connected to a resistive voltage divider referenced to the virtual ground and to relay (1), whose function is to select the attenuation level of the signal before it is sent to the analog-to-digital converter. The VATT_CTRL (Voltage Attenuation Control) pin actuates this relay, selecting either the direct measurement of the subsurface voltage or the measurement of the same voltage attenuated by a factor of 5.

On the other hand, Fig. 3(f) illustrates the input stage dedicated to measuring the injected current, which is based on the use of a precision 22Ω shunt resistor with a tolerance of 0.1%. Under normal operating conditions, this resistor is permanently connected in series with the input of the voltage source responsible for injecting current into the subsurface. However, to prevent parasitic currents or other undesired effects from affecting the measurements, the shunt resistor is not permanently enabled for current measurement; instead, its activation is controlled by a G6K-2F-Y 5VDC relay.

### 2.5 Polarity reversal circuit

**Fig. 4:**
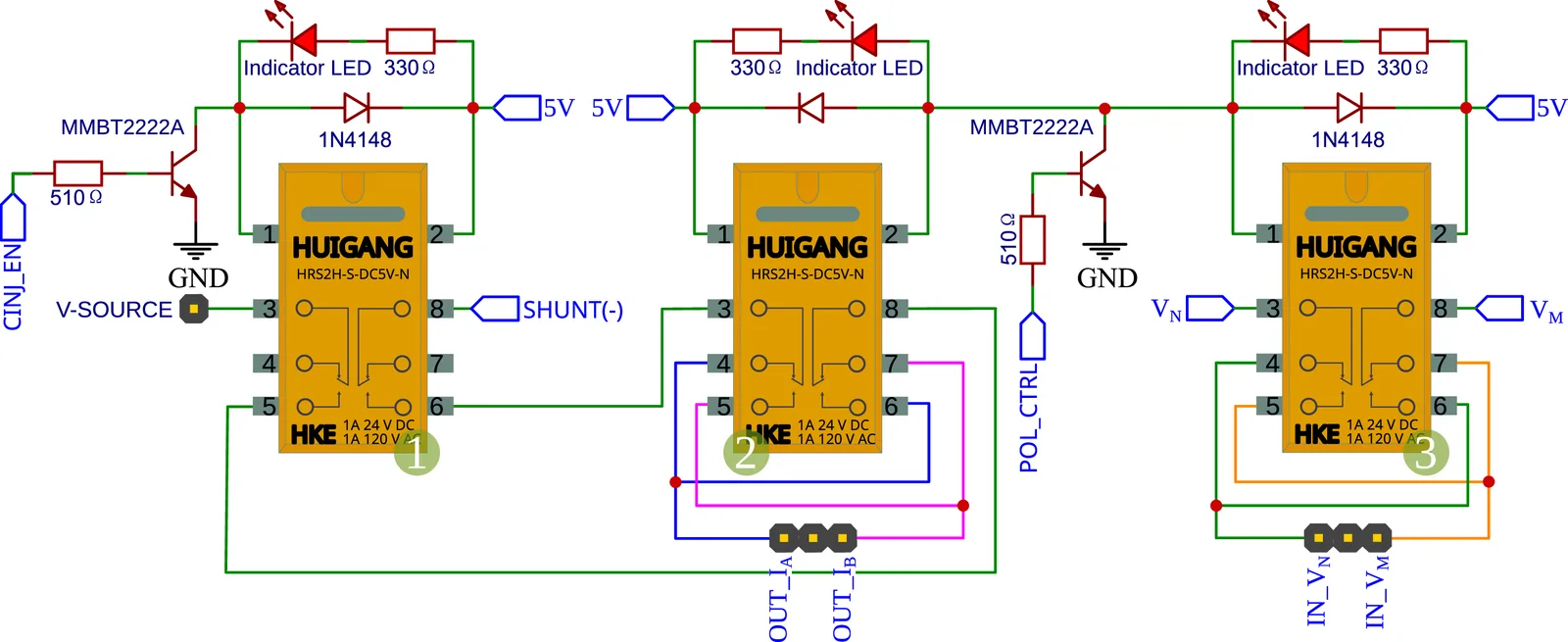
AB current injection and polarity reversal control circuit.

**Fig. 5:**
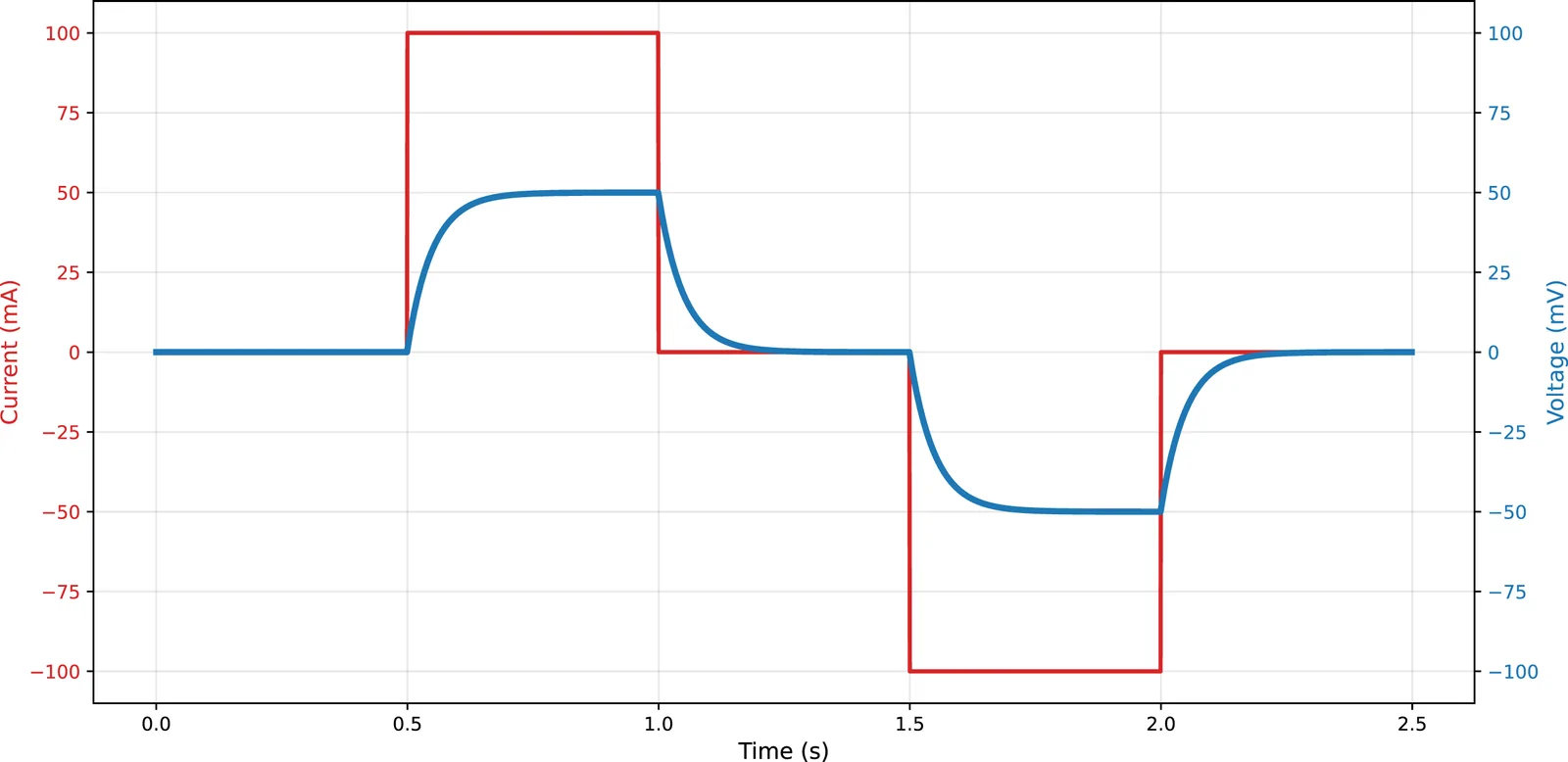
Simulated current sequence established by a voltage source and transient voltage response of the soil for a specific subsurface resistance. The figure illustrates a particular experimental condition in which the applied voltage produces current levels of approximately 100 mA. Following each current reversal, the voltage generated across the measurement electrodes exhibits a transient response and gradually approaches its steady-state value. This behavior requires a waiting period before the voltage is sampled by the ADC to ensure that the measured value corresponds to the stabilized electrical response of the soil.

The circuit shown in Fig. 4 is responsible for injecting the current into the subsurface and reversing its polarity through electrodes A and B. Its operating principle is based on performing two current injections at each measurement point: one in the forward direction and the other in the reverse direction, while measuring the resulting voltage in the ground for both cases. Fig. 5 shows the simulated current injection sequence and the corresponding transient voltage response of the soil. The current is first injected in one direction (+100mA) and then in the opposite direction (−100mA), with a zero-current interval between injections. Following each current reversal, the measured voltage gradually approaches its steady-state value due to the transient electrical response of the soil. The voltage measured during each injection is the superposition of the resulting voltage $V_{res}$ generated by the injected current and the spontaneous potential $V_{esp}$ naturally present in the subsurface. Thus, when the current is injected in one direction, the measured voltage is given by $V_{\to }=V_{res}+V_{esp}$, whereas reversing the current direction yields a measured voltage of $V_{\leftarrow }=V_{res}-V_{esp}$. Summing both measurements gives $V_{m}=2V_{res}$, from which the resulting voltage is obtained as $V_{res}=\frac{1}{2}V_{m}$. To ensure that the resulting voltage recorded by the system is always positive, the polarity reversal of the current electrodes A and B is performed simultaneously with the polarity reversal of the potential electrodes M and N.

Relay (1) isolates the voltage source from the subsurface, ensuring that current injection is performed only when required by the system. The relay is activated by a pulse sent from the microcontroller through the CINJ_EN (Current Injection Enable) pin to the base of the transistor, driving it into saturation and energizing the relay coil.

Once current injection has been enabled, relay (2) determines the direction of current flow. In its normally closed state, the current flows in one direction. However, when a pulse is applied from the microcontroller to the transistor base through the POL_CTRL (Polarity Control) pin, the transistor enters saturation, energizes the coil of relay (2), and reverses the polarity of the injected current. Simultaneously, the same pulse activates relay (3), ensuring that, at the same time the polarity of the current electrodes is reversed, the polarity of the potential electrodes is also reversed. This mechanism guarantees that the resulting voltage measured by the system always maintains a positive polarity, regardless of the current injection direction.

### 2.6 User interface

**Fig. 6:**
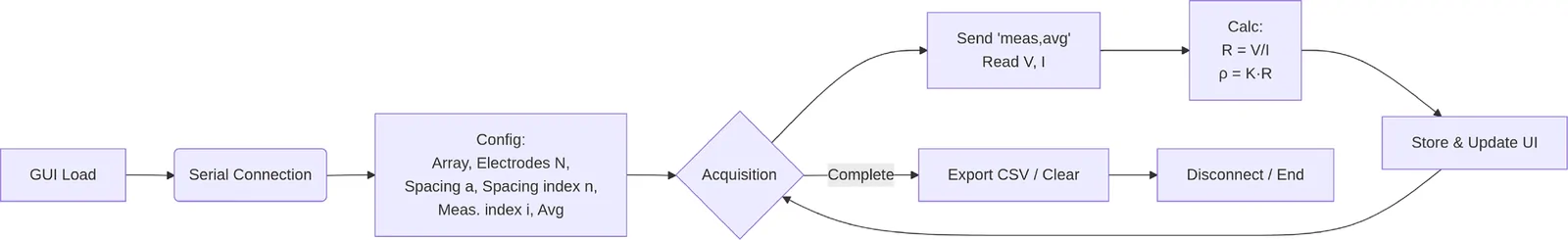
Flowchart of the graphical user interface logic and the data processing pipeline.

**Fig. 7:**
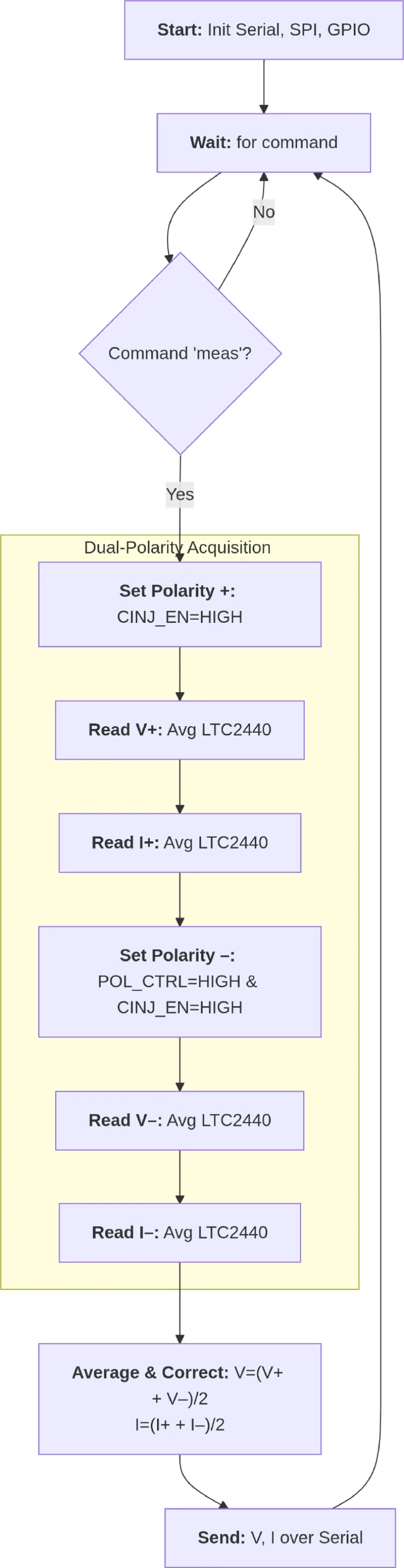
Flowchart of the microcontroller firmware routine for electrical resistivity acquisition using polarity reversal.

The data acquisition system implements a master–slave architecture, in which the browser-based graphical user interface acts as the master, while the microcontroller executes the analog data acquisition routines. The interface establishes a full-duplex communication channel through the serial protocol (Web Serial API on the frontend and hardware UART on the backend), operating at 115 200 baud. The interaction between these two subsystems is illustrated in the flowcharts shown in Fig. 6, Fig. 7.

On the GUI side — Fig. 6 — the user configures the geometric parameters of the electrode array (Wenner, Schlumberger, or Dipole-Dipole), the unit electrode spacing a, the number of electrodes, and the averaging cycles. Once the acquisition is started, the GUI sends the formatted command (‘meas,avg’) through the serial communication link. Upon receiving the voltage (V) and current (I) measurements, the frontend immediately computes the resistance R=V/I, the geometric factor K (determined by the selected array configuration and the spacing index n), the apparent resistivity ρ=K⋅R, and the spatial coordinate of the first electrode X. These values are displayed on the digital multimeter panel and appended to the historical measurement log, while also allowing batch export to CSV format for post-processing.

On the firmware side (microcontroller) — Fig. 7 — the main loop continuously monitors the serial port for incoming commands. Upon receiving the ‘meas’ command, a polarity-reversal measurement sequence is executed to discriminate the spontaneous potential present in the subsurface. The firmware configures the multiplexing relays for the voltage and current measurement paths and communicates with the 24-bit LTC2440 analog-to-digital converter through the SPI peripheral. Successive readings are acquired, discarding the first conversion (to avoid transient effects) and applying linear correction factors over the operating range. This procedure is repeated for both the positive (＋) and negative (−) current polarities, after which the corresponding measurements are arithmetically averaged to obtain the final values of V and I, which are then transmitted back to the GUI.

**Potential hardware applications**

• Development of low-cost resistivity meters for geophysical, geotechnical, and hydrogeological research.
• Implementation of educational platforms for teaching geoelectrical methods and experimental data acquisition.
• Integration into automated multi-electrode systems for electrical resistivity tomography (ERT).
• Evaluation and validation of new polarity-reversal strategies and noise reduction techniques for geoelectrical measurements.
• Adaptation of the platform to other low-amplitude analog signal acquisition applications requiring high resolution.
• PRISM can also be applied in materials physics to implement four-point probe measurements for determining the electrical resistivity of materials such as graphene and other two-dimensional materials [8], perovskite thin films [9], and thermoelectric materials such as bismuth telluride and skutterudites [10].

## 3 Design files summary

**Table:** 

Design filename	File type	Open source license	Location of the file

Hardware schematic	PDF	CERN-OHL-S-2.0	https://doi.org/10.17605/OSF.IO/AUPMJ

Hardware PCB	PDF	CERN-OHL-S-2.0	https://doi.org/10.17605/OSF.IO/AUPMJ

EasyEDA files	ZIP	CERN-OHL-S-2.0	https://doi.org/10.17605/OSF.IO/AUPMJ

Manufacturing files	GBR, BOM, CPL	CERN-OHL-S-2.0	https://doi.org/10.17605/OSF.IO/AUPMJ

GUI	HTML, CSS, JS	GPL-3.0	https://doi.org/10.17605/OSF.IO/AUPMJ

Firmware	INO	GPL-3.0	https://doi.org/10.17605/OSF.IO/AUPMJ

1520 Protector Case	DXF, STEP, PDF, SVG	N/A (Proprietary - Pelican Products, Inc. Terms Apply)	https://doi.org/10.17605/OSF.IO/AUPMJ

**Description of design files**

• **Hardware schematic:** Complete electrical schematic of the proposed hardware, providing the information required to understand, reproduce, and modify the electronic design.
• **Hardware PCB:** Printed circuit board layout of the proposed hardware, including all information necessary for PCB fabrication and assembly.
• **EasyEDA files:** Original EasyEDA project files containing the editable schematic, PCB layout, footprints, and project configuration.
• **Manufacturing files:** Manufacturing files exported from the PCB design, ready for fabrication by standard PCB manufacturers.
• **GUI:** Web-based graphical user interface developed with HTML, CSS, and JavaScript for hardware control, data acquisition, visualization, and data export through the Web Serial API.
• **Firmware:** Arduino firmware (.ino) for the ATmega32U4 microcontroller that controls relay switching, communication with the ADC, and the serial interface.
• **1520 Protector Case:** Collection of the technical files provided by Pelican for the 1520 Protector Case, including the original CAD models, engineering drawings, and vector files available from the manufacturer’s website.

## 4 Bill of materials summary

**Table:** 

Designator	Component	Number	Cost per unit - currency	Total cost - currency	Source of materials	Material type

Microcontroller	Arduino Pro Micro	1	$22.50 USD	$22.50 USD	Sparkfun Electronics	Other

Resistance	Resistor SMD 1206	23	$0.33 USD	$7.59 USD	Mouser	Other

Potentiometer	Multiturn Trimmer Potentiometer	2	$1.89 USD	$3.78 USD	Mouser	Other

Decoupling capacitors	SMD Capacitors 1206	25	$0.401 USD	$10.03 USD	Mouser	Ceramic

Decoupling capacitors	CASE-E_7343	10	$10.48 USD	$104.80 USD	Mouser	Other

Measurement enable relays	Relay G6K-2F-Y-DC5	4	$4.88 USD	$19.52 USD	Mouser	Other

Current and voltage polarity reversal relays	HRS2H-S-DC5V-N	3	$45.16 USD	$135.48 USD	Amazon	Other

ADC	LTC2440	1	$19.02 USD	$19.02 USD	Mouser	Other

Reference voltage	LT1236-5	1	$10.41 USD	$10.41 USD	Mouser	Other

Buffer and virtual ground	Operational LTC2051	1	$9.83 USD	$9.83 USD	Mouser	Other

Buffer	Operational LT1007	1	$7.96 USD	$7.96 USD	Mouser	Other

Shunt resistance	Resistor 22Ω, 1 W ±0.1%	1	$0.40 USD	$0.40 USD	Mouser	Other

Transistor switch	MMBT2222A	6	$0.10 USD	$0.60 USD	Mouser	Other

Flyback diode	1N4148	7	$0.10 USD	$0.70 USD	Mouser	Other

Rectifier diode	1N4007	2	$0.20 USD	$0.40 USD	Mouser	Other

General voltage regulator	LM7815	1	$1.86 USD	$1.86 USD	Mouser	Other

Voltage regulators for the ADC and G6K-2F-Y-DC5 Relays	AMS1117-5	2	$0.70 USD	$1.40 USD	Mouser	Other

HRS2H-S-DC5V relay regulator	78M05	1	$0.42 USD	$0.42 USD	Mouser	Other

Indicator LEDs	LED 0805 RED	10	$0.352 USD	$3.52 USD	Mouser	Other

Case	1520 protector case	1	$204.95 USD	$204.95 USD	Pelican	Other

## 5 Build instructions

The acquisition module was developed from the circuit schematics and printed circuit board (PCB) layout designed using the open-source software EasyEDA. The source files for the schematic, PCB layout, and manufacturing files are available in the *Design files summary* section, while the complete list of components, commercial part numbers, and associated costs is provided in the *Bill of materials summary* section.

**Fig. 8:**
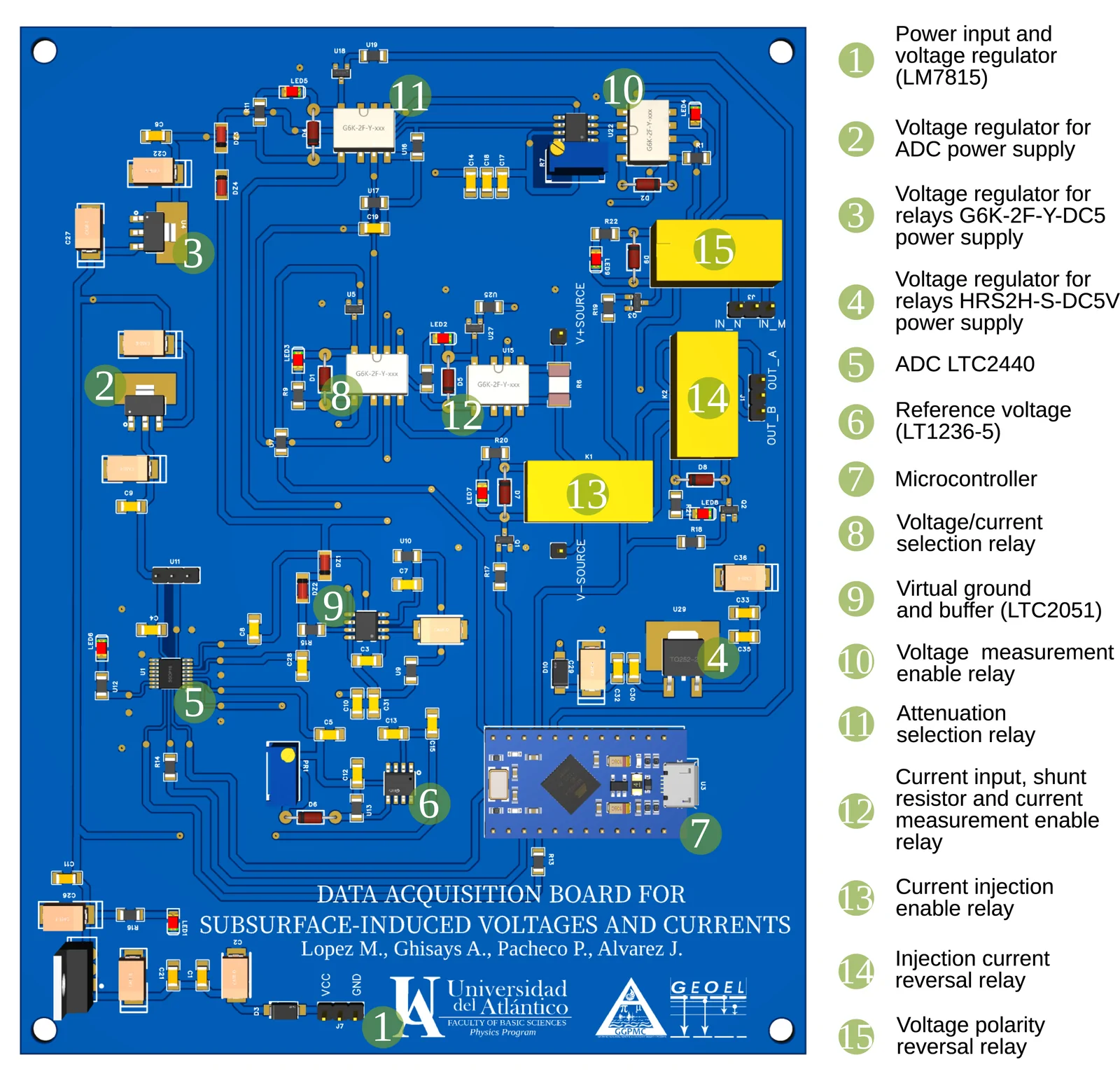
PCB data acquisition board for injected currents and resulting voltages in the subsurface.

The PCB was manufactured by JLCPCB, which offers the option of supplying the boards with the surface-mount device (SMD) components pre-assembled. However, to facilitate design validation, inspection of solder joints, and component replacement during the development stage, the board was assembled manually in the laboratory. Fig. 8 shows the fully assembled PCB, where the main functional blocks of the system can be identified, including the power supply stage, the voltage reference, the 24-bit analog-to-digital converter, the virtual ground circuit, the microcontroller, and the different relay-based switching stages used for current injection, polarity reversal, gain selection, and voltage and current measurement enable.

The assembly process consisted of soldering the SMD components using solder paste and hot air, followed by the installation of the through-hole technology (THT) components. Subsequently, the board was inspected to verify the absence of short circuits and the correct orientation of polarized components, particularly voltage regulators, diodes, integrated circuits, electrolytic capacitors, and relays. Before installing the microcontroller and the analog-to-digital converter, the supply voltages and voltage references were verified at the corresponding test points.

During assembly and commissioning, the standard precautions required for precision electronic circuits should be observed. It is recommended to verify the polarity of the power supply before energizing the board, visually inspect all solder joints, check for the absence of short circuits between the power and ground planes, and perform the initial power-up using a laboratory power supply with current limiting enabled. These verification procedures minimize the risk of damaging high-precision components and ensure the correct operation of the acquisition module before its integration into the complete system.

The source code of the graphical user interface, together with the installation and execution instructions, is available in the table included in the *Design files summary* section. The application was developed as a web-based tool and must be executed in a browser compatible with the Web Serial API, such as Google Chrome, Microsoft Edge, or Opera, allowing direct serial communication with the acquisition hardware without requiring additional software.

**Fig. 9:**
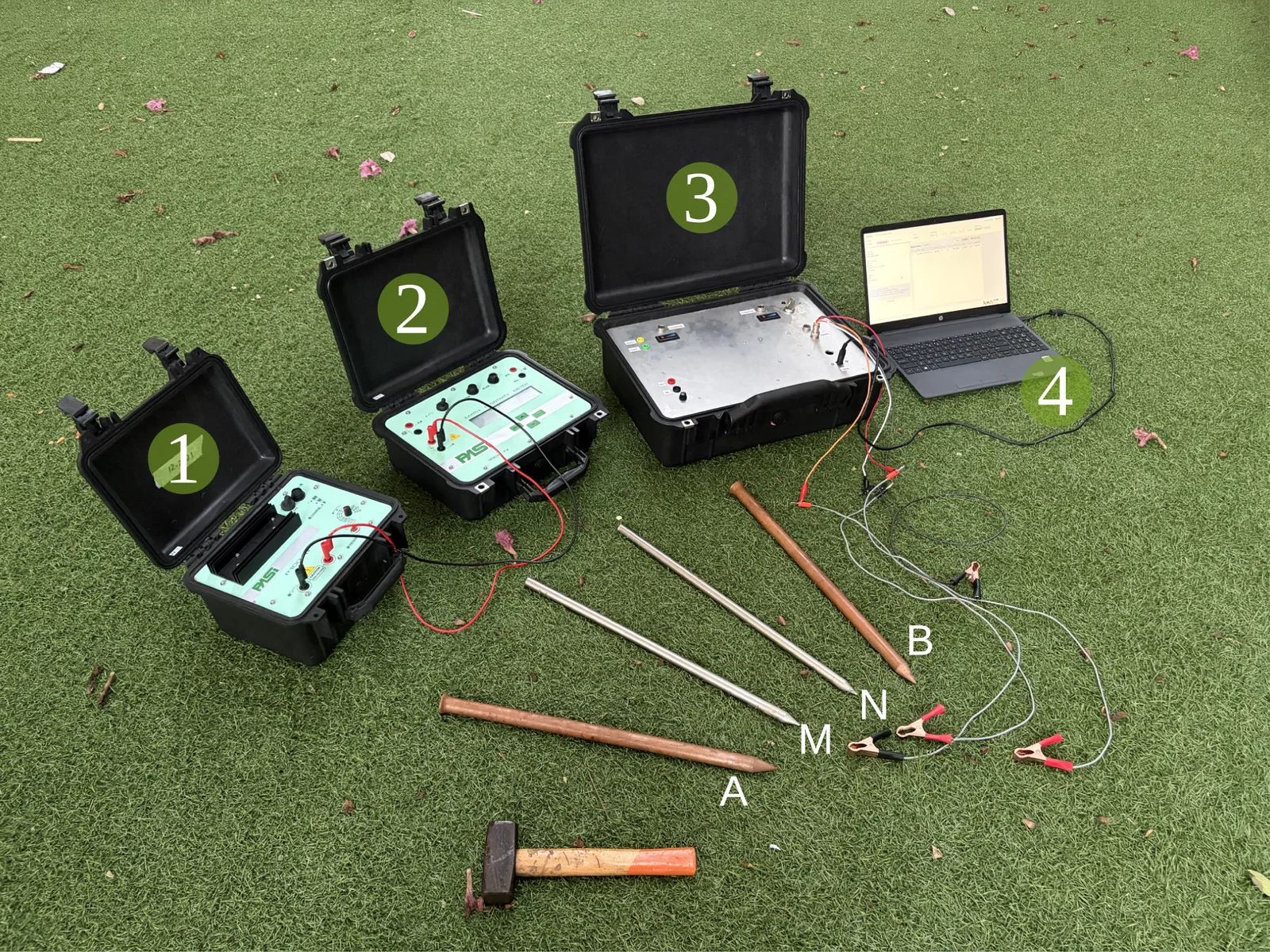
Experimental setup for electrical resistivity data acquisition, including the developed system, the commercial reference instrument, and a four-electrode (ABMN) measurement configuration representative of geoelectrical surveys.

Fig. 9 shows a commercial instrument and the developed system. Items (1) and (2) correspond to a PASI P100-2 voltage source and a PASI Earth Resistivity Meter MOD. 16GL-N, respectively. This commercial system is used for the same purpose as the developed hardware; however, its acquisition stage is based on a 16-bit ADC.

Items (3) and (4), on the other hand, correspond to the developed acquisition module and the graphical user interface running on a laptop computer, respectively. The figure also shows the four terminals A, B, M, and N, which connect the acquisition module to the electrodes installed in the ground.

During the data acquisition campaigns, the voltage source (1), the acquisition module (3), and the graphical user interface (4) constitute the complete electrical resistivity measurement system. Subsequently, in the **Validation and characterization** section, an experimental laboratory comparison between the proposed system and the commercial instrument is presented in order to evaluate the performance and consistency of the acquired measurements.

It should be noted that the present work focuses exclusively on the development and laboratory characterization of the proposed hardware platform. Although the system has been designed for electrical resistivity surveys using standard four-electrode configurations, its performance under real field survey conditions has not been evaluated in this study. A comprehensive field validation, including the assessment of the system in practical geophysical surveys and comparison with commercial instruments under operational conditions, is considered a subject for future work.

### 5.1 Automated PCB assembly

The PCB was designed to support commercial SMT assembly services. All manufacturing files required for automated assembly are included in the repository.

The required files are:

• Gerber files for PCB fabrication.
• Bill of Materials (BOM) containing manufacturer part numbers and component references.
• Component Placement List (CPL/Centroid) specifying the position, rotation, and side of every surface-mount component.

These files can be directly uploaded to PCB manufacturers such as JLCPCB or equivalent assembly services, allowing the complete surface-mount assembly of the board prior to shipment. After receiving the assembled PCB, only the through-hole components must be soldered manually before system verification.

### 5.2 Manual laboratory assembly

For laboratory prototyping, the board can be assembled manually using solder paste, hot air rework equipment, and conventional soldering tools. The recommended assembly sequence is:

1. Inspect the manufactured PCB for fabrication defects.
2. Apply solder paste to all SMD pads.
3. Solder the voltage regulators and power supply circuitry.
4. Solder the precision voltage reference and the virtual ground circuit.
5. Solder the critical integrated circuits, including the LTC2440 ADC, operational amplifiers, and logic devices.
6. Solder the remaining passive SMD components.
7. Inspect all solder joints using magnification and verify the absence of bridges.
8. Power the board using a current-limited laboratory power supply and verify all supply voltages before installing sensitive integrated circuits whenever possible.
9. Solder all through-hole components, including relays, connectors, pin headers, terminal blocks, and electrolytic capacitors.
10. Install the ATmega32U4 microcontroller.
11. Verify continuity between power and ground planes.
12. Load the firmware and perform the calibration procedure described in the Validation and Characterization section.

## 6 Operation instructions

System operation begins by connecting the four measurement terminals (A, B, M, and N) to the electrodes installed in the ground according to the selected electrode configuration. Subsequently, the power supply is connected, and serial communication between the acquisition module and the computer is established through a USB cable (Fig. 9). The GUI is executed in a Web Serial API-compatible browser, from which the corresponding serial port is selected and communication with the instrument is initiated.

**Fig. 10:**
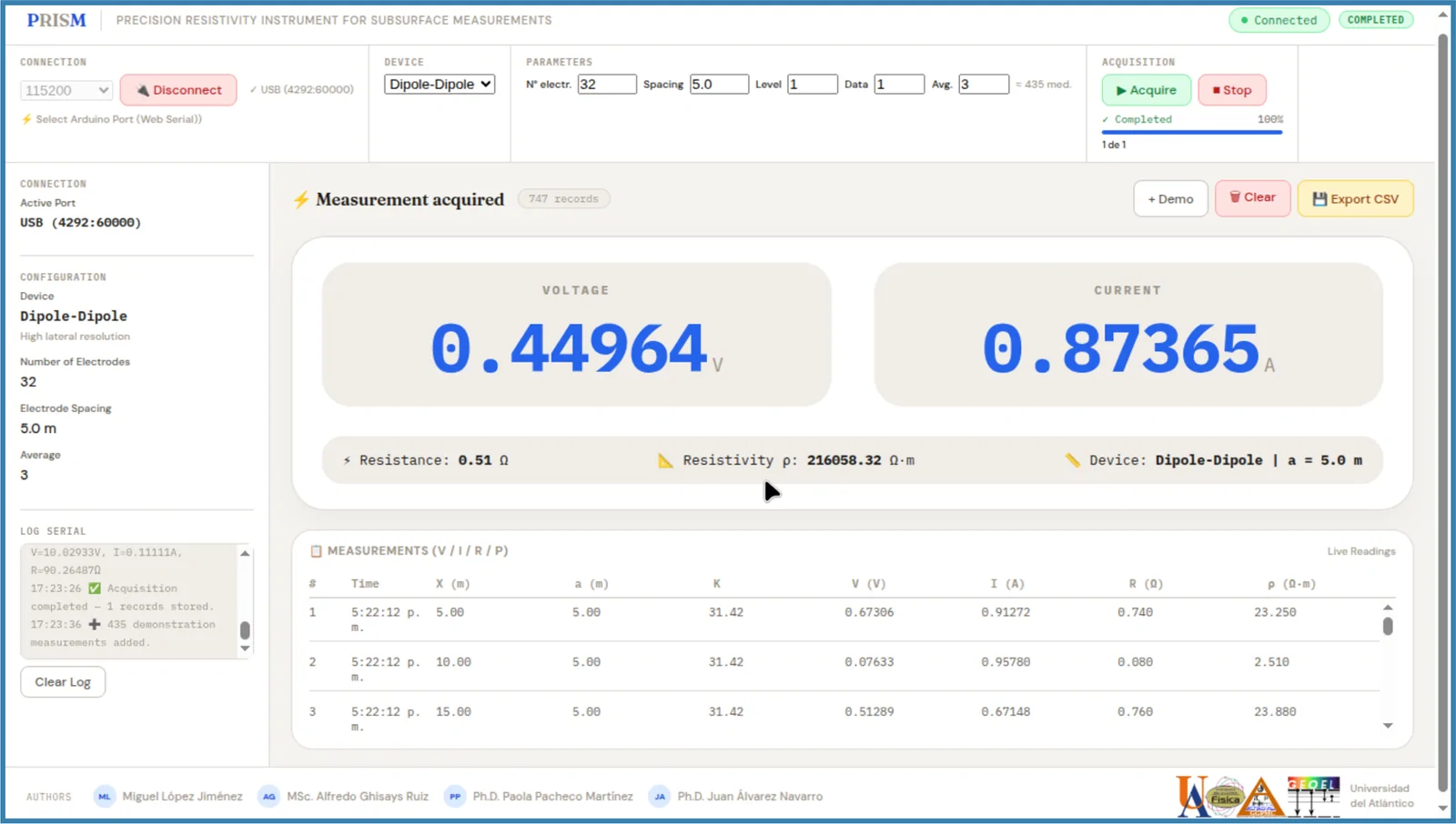
Graphical user interface used to control the data acquisition process.

Once the connection has been established, the user configures the acquisition parameters (Fig. 10) and starts the measurement process. The GUI transmits the acquisition command to the microcontroller, which controls the relay switching sequence, reverses the current polarity whenever required, and acquires the voltage and current measurements using the ADC. The acquired data are displayed in real time on the GUI and can be stored for subsequent processing and inversion.

Before starting a measurement campaign, it is recommended to verify the continuity of the cables, the correct connection of the electrodes, and the stability of the power supply. Likewise, the electrode connections should not be modified while current injection is active in order to avoid electrical transients that could affect the integrity of the measurements or damage the acquisition electronics. Although the system operates at low voltage and current levels, all electrical connections should be made with the power supply turned off and in accordance with the basic safety practices for electronic instrumentation equipment.

## 7 Validation and characterization

Once the system had been assembled, calibration and characterization procedures were carried out, beginning with the construction of voltage and current calibration curves using a 5$\frac{1}{2}$-digit Siglent SDM3055 digital multimeter as the reference instrument.

For the voltage calibration, a precision calibrated voltage reference based on the LT1021 integrated circuit was used to generate known and stable voltage values for evaluating the system response. For the current calibration, a Newport Model 505 precision laser diode current source was employed due to its high stability during operation.

### 7.1 Characterization and calibration of voltage and current measurements

Using the 10 V output of the voltage reference, a resistive voltage divider was implemented, consisting of three 2kΩ resistors connected in series, followed by three 510Ω resistors also connected in series, all with a tolerance of 1%. This configuration provided eight different voltage levels: four above 2 V and four below 2 V. These voltages were measured using the reference multimeter, selecting the 2 V range for voltages below this threshold and the 20 V range for higher voltages, thereby ensuring the maximum resolution for each measurement.

Using the voltage values measured with the reference multimeter and the corresponding digital values obtained from the system ADC at a conversion rate of 6.9Hz, linear regression was performed to obtain the calibration equations.

**Fig. 11:**
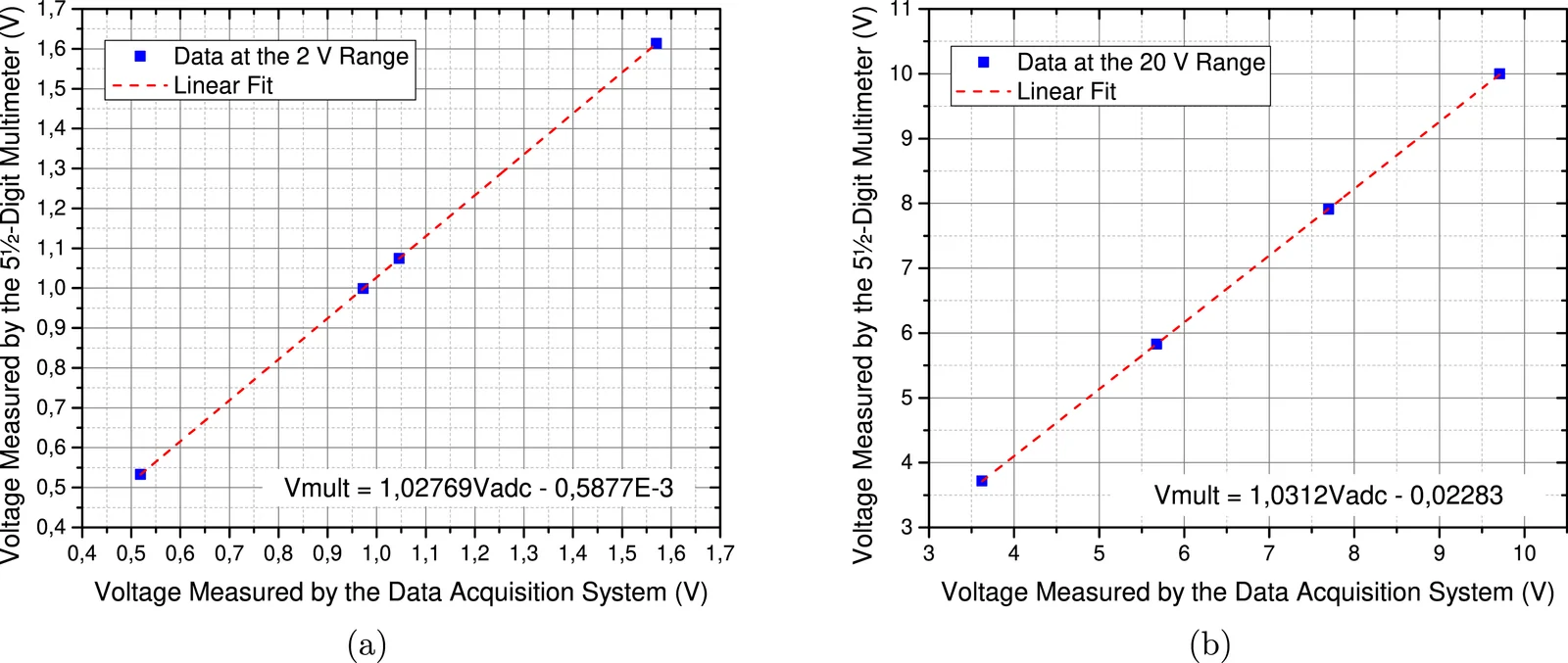
Calibration curves for voltage measurements over the two operating ranges. (a) Calibration curve for voltage measurements below 2 V. $R^{2}=0.9999$. Δm=0.00038. Δb=0.0004185 V. (b) Calibration curve for voltage measurements between 2 V and 10 V. $R^{2}=0.9999$. Δm=0.0024. Δb=0.01672 V.

Due to the different measurement ranges of the reference multimeter and the developed system (Fig. 3(g)), two independent calibration curves were required to achieve proper linearization. Fig. 11(a) presents the calibration curve and the corresponding regression equation for voltage measurements in the range from 0 to 2 V. Likewise, Fig. 11(b) shows the calibration curve for input voltages between 2 V and 10 V. These two calibration equations were ultimately implemented in the firmware to ensure accurate voltage measurements over the entire operating range of the instrument.

Following the same procedure used for the characterization of the voltage measurements, a calibration process was carried out for the current measurements to ensure the accuracy and reliability of the data acquired by the developed system.

Considering the current range to be characterized, from 0 to 100 mA, two measurement ranges were selected to maximize the resolution and accuracy of the readings: one for currents below 10 mA and another for currents between 10 mA and 100 mA. For each range, ten measurement points were acquired over the corresponding interval.

Based on the acquired data, the relationship between the current values measured by the system and those measured by the reference multimeter was established by applying linear regression. This procedure made it possible to determine the calibration equations required to correct the system measurements for each operating range.

**Fig. 12:**
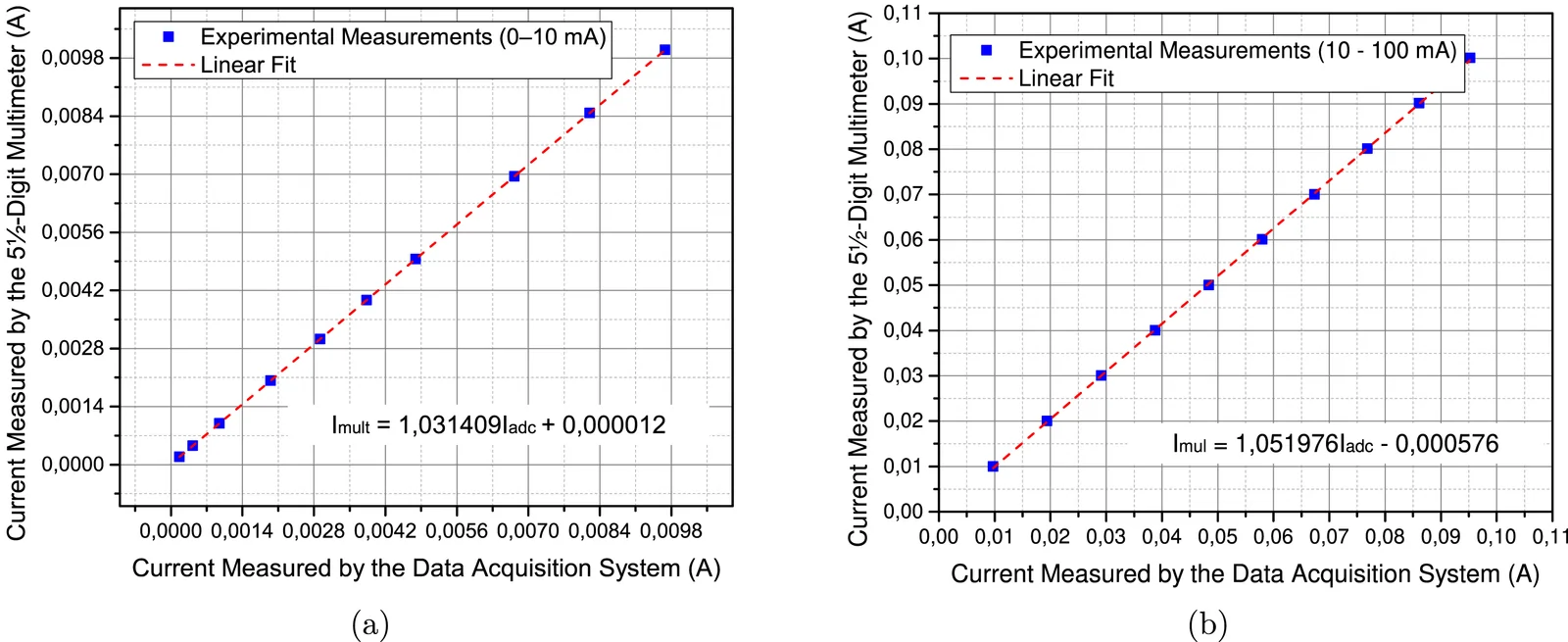
Calibration curves for current measurements over the two operating ranges. (a) Calibration curve for current measurements in the range from 0 mA to 10 mA. $R^{2}=0.9999$. Δm=0.000081. $\Delta b=4\times 10^{-7}$ A. (b) Calibration curve for current measurements in the range from 10 mA to 100 mA. $R^{2}=0.9999$. Δm=0.003468. Δb=0.000206 A.

Fig. 12(a) presents the calibration curve corresponding to the low-current range, from 0 to 10 mA. Within this interval, a well-defined linear relationship is observed between the compared quantities, enabling accurate correction of the measurements under low-current conditions. Similarly, Fig. 12(b) presents the calibration curve for the intermediate current range, from 10 mA to 100 mA, where a consistent linear behavior is likewise observed.

The calibration equations obtained from this characterization procedure were subsequently implemented in the firmware of the master microcontroller, enabling the system to automatically select the appropriate calibration function according to the detected current range. This approach ensures accurate measurements over the entire operating range of the instrument, contributing to the consistency and reliability of the acquired data.

### 7.2 Evaluation of the repeatability, stability, and resolution of the data acquisition system

After loading the calibration equations for each voltage and current range into the microcontroller firmware, the repeatability, stability, and intrinsic resolution of the ADC were evaluated. For this purpose, a statistical test was performed by acquiring 10,000 consecutive measurements of two highly stable reference voltages: 0.999091 V and 10.000000 V, generated by the calibrated precision voltage reference. It is important to emphasize that this analysis was not intended to validate the absolute accuracy of the calibration equations, but rather to characterize the ADC performance in terms of repeatability, measurement dispersion, and noise. These characteristics are directly applicable to the current measurements, since the ADC ultimately measures the voltage drop across the shunt resistor.

**Fig. 13:**
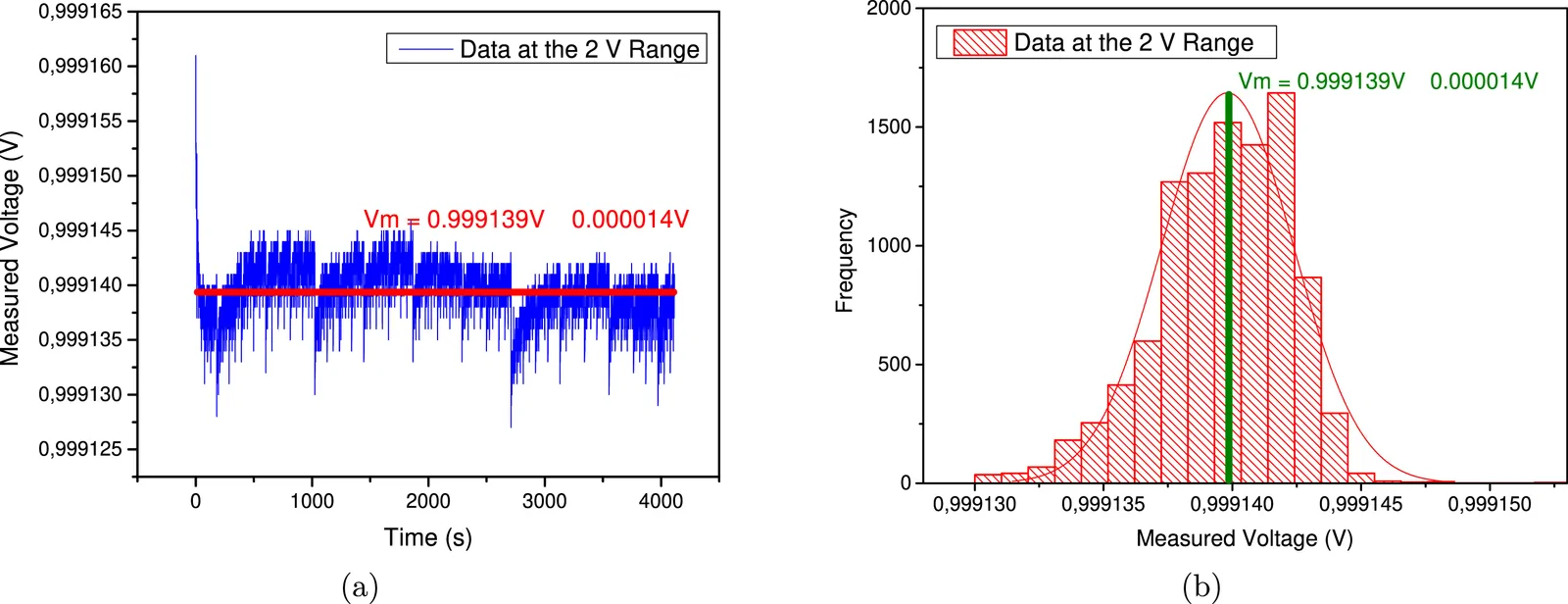
Statistical evaluation of the acquisition system using the 0.999091 V precision voltage reference. (a) Reference voltage measured 10,000 times using the acquisition system in the 2 V range. $RMS\;noise=2.5\times 10^{-6}$ V. $pk\text{-}pk\;noise=3.4\times 10^{-5}$ V. ENOB=19 bits. (b) Distribution of the reference voltage measured 10,000 times using the acquisition system in the 2 V range.

The 10,000 measurements of the 0.999091 V reference voltage are shown in Fig. 13(a). The mean value obtained for this measurement series was 0.999139 V, with a standard deviation of 2.57μV and an uncertainty of 14μV, corresponding to a percentage error of only 0.0048% with respect to the calibrated reference value. The measurement exhibits remarkable stability, with consistent readings up to the fourth decimal place, while variations appear only in the fifth decimal place, which therefore represents the uncertain or noise digit.

This level of dispersion, together with the stability observed in the measurement series, indicates that the system is capable of reliably resolving and measuring signals on the order of 100μV, since these signals are well above the measurement noise and uncertainty. Consequently, the effective resolution of the acquisition system is not determined solely by the nominal resolution of the converter but also by the combined contributions of noise and uncertainty introduced by the electronic components implemented throughout the measurement chain.

The effective number of bits (ENOB) was calculated using Eq. (3), where N (bits) is the nominal resolution of the analog-to-digital converter, ${\text{Noise}}_{rms}$ (V) is the measured root-mean-square noise at the converter output, and $V_{ref}$ (V) is the reference voltage. The ENOB provides an estimate of the actual resolution of the converter by accounting for the degradation caused by noise and other non-ideal effects, thereby indicating the equivalent number of bits of an ideal ADC that would exhibit the same RMS noise performance [11].

(3) $$ENOB=N-\log_{2}\left(\frac{2^{N}}{V_{ref}}{\text{Noise}}_{rms}\sqrt{12}\right)$$

Additionally, Fig. 13(b) shows the distribution of the measured values, which closely follows a Gaussian distribution. This behavior is characteristic of random processes such as thermal electrical noise and confirms that the observed fluctuations arise from the intrinsic uncertainty of the conversion process rather than from systematic drift.

**Fig. 14:**
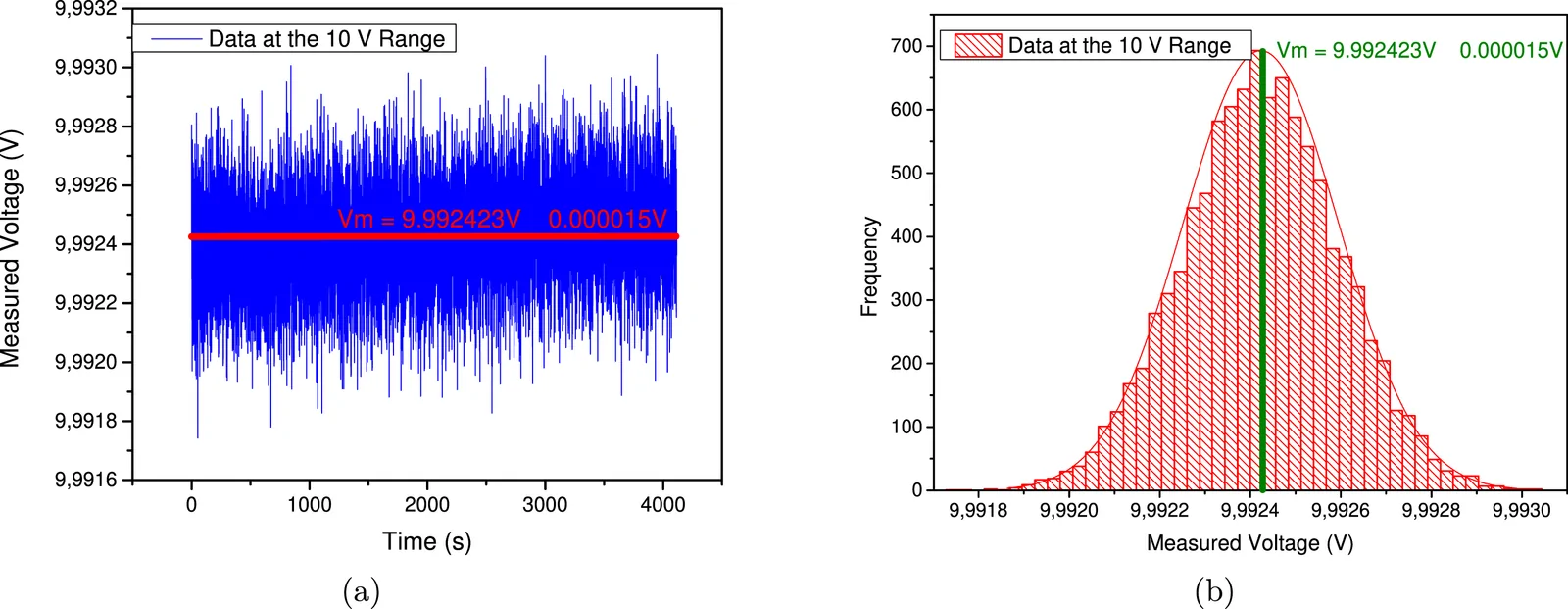
Statistical evaluation of the acquisition system using the 10.000000 V precision voltage reference. (a) Reference voltage measured 10,000 times using the acquisition system in the 10 V range. RMSnoise=0.000172 V. pk-pknoise=0.001302 V. ENOB=13 bits. (b) Distribution of the reference voltage measured 10,000 times using the acquisition system in the 10 V range.

An analogous analysis was performed for the high-voltage measurement range. Fig. 14(a) presents the 10,000 measurements of the 10.000000 V reference voltage.

In this case, the mean measured value was 9.992423 V, with a standard deviation of 172μV and an uncertainty of 15μV, corresponding to a percentage error of 0.076% relative to the calibrated reference value. High stability is observed up to the third decimal place, whereas increased dispersion appears from the fourth decimal place onward, defining the uncertain digit of the measurement. This behavior can be primarily attributed to the resistive voltage divider with an attenuation factor of five used to adapt voltages above 2 V to the ADC input range. The resistor tolerances and associated thermal noise introduce additional fluctuations into the measured signal. Nevertheless, the measurement distribution shown in Fig. 14(b) exhibits a clear Gaussian profile, indicating that these fluctuations are dominated by random effects rather than uncontrolled systematic errors.

In contrast, the 0.999091 V signal (Fig. 13(a)) exhibits significantly greater stability because it bypasses the resistive divider and is conditioned only by the operational amplifiers before being digitized by the ADC. This substantially reduces additional noise sources, resulting in lower measurement dispersion.

Overall, these results demonstrate that the acquisition system provides high stability, repeatability, and resolution, enabling reliable measurements in the microvolt range, particularly for signals above approximately 100μV. They also show that the signal-conditioning architecture, especially the use of resistive voltage dividers for high-voltage measurements, introduces additional limitations that must be considered when evaluating the overall performance of the system.

Since current measurements are performed indirectly by measuring the voltage drop across a shunt resistor, these conclusions regarding ADC stability and performance are directly applicable to the current measurements, thereby validating the integrity of the complete acquisition system.

The use of a 22Ω shunt resistor imposes an upper limit on the measurable current range. Since the ADC accepts a maximum input voltage of approximately 2.5 V, the maximum measurable current is given by

(4) $$I_{max}=\frac{2.5\;\text{V}}{22\;\Omega }\approx 113.6\;\text{mA}.$$

In practice, this value is conservatively limited to approximately 100mA as the maximum operating current. Consequently, the system is capable of measuring currents over an approximate range from 100μA to 100mA. This operating range is well suited for field applications, since the injected currents typically employed in geoelectrical surveys rarely exceed 100mA. Therefore, the available operating range adequately covers the practical conditions encountered during field data acquisition.

Furthermore, the system allows the experimental conditions to be adjusted by varying the voltage supplied by the voltage source that injects current through electrodes A–B. This enables control of the injected current magnitude, optimizing the signal-to-noise ratio while ensuring that the voltage drop across the shunt resistor remains within the dynamic input range of the ADC. Consequently, the integrity of the measurements is preserved without compromising the resolution or accuracy of the acquisition system.

### 7.3 Evaluation of the dynamic range of the data acquisition system and comparison with a commercial instrument

To evaluate the dynamic range of the acquisition system, controlled measurements were performed using precision resistors with a tolerance of 1%. The experimental setup was designed to approximately reproduce the measurement conditions encountered during field geoelectrical data acquisition.

Under these conditions, measurements were performed on a set of resistors with known nominal values, ranging from 10Ω to 68kΩ. For comparison purposes, the known nominal value of each resistor was considered as the reference value. The current injected through the current electrodes (A–B) was supplied by a *PASI* P100-2 voltage source with an operating range from 0 to 200V. The voltage applied to each resistor was adjusted according to its nominal resistance in order to ensure appropriate measurement conditions. This procedure is analogous to that employed during field surveys, where the excitation level (AB) is adjusted according to the electrical characteristics of the subsurface.

**Fig. 15:**
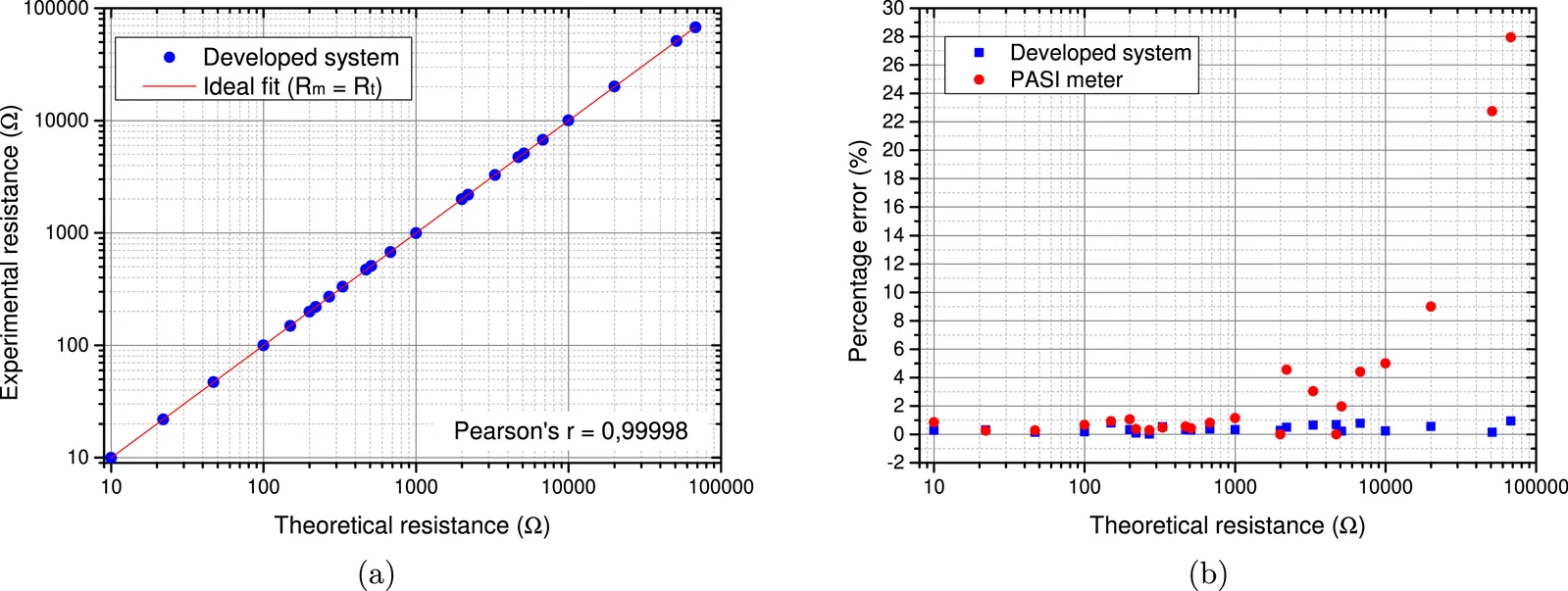
Comparison of the resistance measurement performance of the developed system and the commercial instrument over the evaluated dynamic range. (a) Comparison between the measured and known resistance values. The plot illustrates the agreement between the experimental measurements and the expected values, where perfect agreement is represented by the line $R_{m}=R_{t}$. MeanRelativeError=0.45%. (b) Comparison of the percentage measurement error for different known nominal resistance values. MeanRelativeError(PASI)=3.77%. MeanRelativeError(PRISM)=0.39%.

From the experimental measurements performed on the set of test resistors, the measured resistance values ($R_{m}$) were calculated using Ohm’s law from the measured voltage ($V_{m}$) and injected current ($I_{m}$). These results were then used to construct Fig. 15(a), which evaluates the performance of the developed acquisition system over the investigated dynamic range.

Fig. 15(a) presents the direct comparison between the measured and known resistance values. The experimental data closely follow the ideal relationship $R_{m}=R_{t}$, demonstrating excellent agreement between the measurements obtained with the developed instrument and the expected resistance values. The dispersion of the data points around the ideal line remains minimal throughout the evaluated range, indicating good linearity and measurement accuracy.

Under identical experimental conditions, the same set of test resistors was measured using the commercial Earth Resistivity Meter PASI MOD. 16GL-N. The relative percentage error with respect to the known nominal resistance values ($R_{t}$) was then calculated for both the commercial instrument and the developed system. The experimental results are presented in Fig. 15(b), which facilitates a direct comparison of the performance of the proposed system with that of the commercial instrument.

The results show that the measurement error of the developed system remains below 1% over several orders of magnitude of resistance. It is particularly noteworthy that, despite the wide resistance range evaluated—from 10Ω to 68kΩ—the maximum recorded percentage error was only 0.95%, which does not exceed the nominal 1% tolerance of the precision resistors used in the experiments.

Furthermore, the random variation of the measurement error indicates that the performance of the developed system does not depend directly on the resistance value itself, but rather on the electrical quantities involved in the measurement, specifically the applied voltage and the resulting current. Since the excitation voltage was individually adjusted for each resistor, an adequate signal-to-noise ratio was maintained throughout the experiments, following the same strategy commonly adopted in field geoelectrical surveys. Overall, these results demonstrate that the proposed acquisition system exhibits reliable and consistent performance throughout the evaluated dynamic range while maintaining measurement errors within the expected limits imposed by the tolerance of the reference components.

In contrast, the error associated with the measurements acquired using the commercial instrument remains, on average, below 1% only up to approximately 2200Ω. Beyond this resistance value, the percentage error increases progressively, reaching approximately 27% for the 68kΩ resistor. This behavior highlights the performance differences between the two measurement systems, which are related to the characteristics of their respective acquisition and signal-processing chains.

In particular, although the commercial Earth Resistivity Meter PASI MOD. 16GL-N employs a 16-bit ADC whereas the developed system incorporates a 24-bit ADC, the improved performance cannot be attributed exclusively to the higher ADC resolution. Rather, it results from the combined contribution of the complete measurement chain, including the high-resolution ADC, the low-noise voltage reference, the analog signal-conditioning circuitry, the PCB design, the firmware implementation, and the automatic polarity-reversal technique. Together, these elements provide higher effective resolution, improved noise immunity, and enhanced measurement stability over a wider dynamic range. Consequently, the developed system achieves more reliable and accurate resistance measurements, particularly under low-amplitude signal conditions commonly encountered in geoelectrical prospecting.

From a geophysical perspective, the resistance range evaluated in the laboratory can be directly related to the electrical resistivity values commonly found in Colombian subsurface materials. In general, clay-rich, highly weathered, or water-saturated soils — typical of alluvial and tropical environments — exhibit low resistivities, on the order of a few to several tens of Ωm. Partially saturated sediments, such as sands and gravels, generally fall within intermediate resistivity ranges, from several tens to several hundreds of Ωm. In contrast, more competent lithologies, including consolidated sandstones, limestones, and some volcanic rocks, may exhibit resistivities ranging from several hundreds to several thousands of Ωm, whereas massive igneous and metamorphic rocks, such as granites, compact basalts, and quartzites, can exceed several thousand Ωm. These lithological units are widely distributed throughout Colombia, as documented by the geological mapping of the Colombian Geological Survey [12].

### 7.4 Hardware capabilities and limitations

Based on the validation results presented earlier, the main operational capabilities and limitations of the PRISM acquisition system are summarized below:

• Bipolar voltage and current measurement from −100μV to −2 V, 100μV to 10 V, and 100μA to 100 mA using automatically selected measurement ranges.
• Analog-to-digital conversion performed using a 24-bit LTC2440 delta-sigma ADC operating at 6.9 samples/s.
• High measurement accuracy and precision were demonstrated through agreement with reference voltages and the repeatability of consecutive measurements under laboratory conditions.
• Automatic relay switching enables selection of measurement range, current injection polarity, and voltage or current acquisition without user intervention.
• Limitation: the instrument is intended exclusively for DC electrical resistivity measurements and is not suitable for AC impedance or induced polarization surveys.
• Limitation: the hardware has been validated using laboratory reference instruments; long-term stability under continuous field operation has not yet been evaluated.
• Limitation: the acquisition speed is optimized for precision measurements rather than rapid data acquisition.

### 7.5 Conclusion

The open-source PRISM data acquisition system offers a low-cost, reproducible solution for direct current (DC) electrical resistivity measurements. Experimental validation demonstrated reliable voltage and current measurements, as well as high linearity, accuracy, and precision within the validated operating ranges. The full release of hardware design files, firmware, software, and assembly documentation facilitates easy replication, customization, and ongoing development by the geophysical and scientific instrumentation communities.

## CRediT authorship contribution statement

**Miguel López Jiménez:** Writing – original draft, Visualization, Validation, Software, Methodology, Investigation, Formal analysis, Data curation. **Alfredo Ghisays Ruiz:** Writing – review & editing, Validation, Supervision, Resources, Project administration, Methodology, Investigation, Funding acquisition, Conceptualization. **Paola Pacheco Martínez:** Writing – review & editing, Validation, Supervision, Resources, Project administration, Methodology, Investigation, Funding acquisition, Conceptualization. **Juan Álvarez Navarro:** Writing – review & editing, Validation, Supervision, Resources, Project administration, Methodology, Investigation, Funding acquisition, Conceptualization.

## Ethics statements

This work did not involve experiments on human participants or animals. No human biological samples, animal specimens, or personal data were collected or analyzed. Therefore, ethical approval and informed consent were not required for this study.

## Declaration of competing interest

The authors declare that they have no known competing financial interests or personal relationships that could have appeared to influence the work reported in this paper.
